# Association of Thrombomodulin Gene C1418T Polymorphism with Susceptibility to Kawasaki Disease in Chinese Children

**DOI:** 10.1155/2018/1064380

**Published:** 2018-06-12

**Authors:** Yapeng Lu, Rui Liu, Luting Zha, Shan Yuan, Lang Tian, Jia Chen, Lihua Huang, Zuocheng Yang

**Affiliations:** ^1^Department of Pediatrics, The Third Xiangya Hospital of Central South University, Changsha, Hunan 410013, China; ^2^Central Laboratory, The Third Xiangya Hospital of Central South University, Changsha, Hunan 410013, China

## Abstract

Kawasaki disease (KD) is an acute systemic vasculitis that predominantly affects children and can result in coronary artery lesions (CALs). Thrombomodulin (TM) is a critical cofactor in the protein C anticoagulant system. The *TM* C1418T (rs1042579) polymorphism is associated with a high risk of cardiac-cerebral vascular diseases. But the association of the *TM* C1418T polymorphism with susceptibility to KD, CAL formation, and intravenous immunoglobulin (IVIG) resistance is still unclear. In our study, we examined the *TM* C1418T polymorphism in 122 children with KD and 126 healthy children and revealed the correlation between the *TM* C1418T polymorphism and KD, CAL formation, and IVIG resistance.

## 1. Introduction

Kawasaki disease (KD) also known as mucocutaneous lymph node syndrome (MCLS) was first described by Tomisaku Kawasaki in 1967. It is characterized by prolonged fever, diffuse mucosal inflammation, indurative edema of the hands and feet, a polymorphous skin rash, and nonsuppurative lymphadenopathy. KD is a multisystem vasculitis that can result in coronary artery lesions (CALs), which are known to predominantly affect young children (84%~86% of cases occur in children between 6 months and 5 years of age) with a male predominance (approximately 1.5~1.8 times higher than females) [1]. The disease is self-limited; patients with KD mostly have a good prognosis with active treatment [2]. Coronary artery involvement is the most common complication of KD and may cause significant coronary stenosis, which results in coronary artery aneurysm and occlusion, leading to myocardial ischemia and infarction lesions. In recent years, the incidence rate of KD has increased [3]. A recent study on the epidemiology of KD described that the disease has replaced rheumatic heart disease as the most common cause of acquired heart disease in children in developed countries and in some developing countries [4]. KD is considered to be a potential risk factor for ischemic heart disease in adults and sudden cardiac death in young adults [5].

The etiopathogenesis of KD has not yet been clearly identified. It is speculated that KD is the result of the interaction among infection, immune dysfunction, and genetic susceptibility. There are significant differences in the morbidity among different racial groups; for example, Asian children are significantly more susceptible [6]. The incidence in siblings of KD patients and in offspring of parents with a history of KD is higher than that of the general population, which indicates that genetic susceptibility may play an important role in the pathogenesis of this disease [7]. In addition, recent research demonstrates that some single-nucleotide polymorphism (SNP) loci of genes regulating immune, inflammation, and blood coagulation, such as the tumor necrosis factor (TNF) gene, interleukin-related genes, and platelet endothelial cell adhesion molecule-1 (PECAM-1), have a close relationship with KD [8–10].

Thrombomodulin is considered a marker of vascular endothelial injury [11]. *TM* gene polymorphisms may be associated with myocardial infarction and cerebral infarction [12, 13], but it is unclear whether there is an association between the pathogenesis of KD and *TM* gene polymorphisms.

The objective of this study is to verify the hypothesis that the *TM* C1418T polymorphism may play a role in the risk assessment of KD. To the best of our knowledge, this is the first study that investigates the role of the *TM* C1418T polymorphism in KD.

## 2. Materials and Methods

### 2.1. Study Subjects

A total of 122 patients with KD were enrolled from January 2012 to December 2016 in the Third Xiangya Hospital, Changsha, China. All patients met the diagnostic criteria of the Japanese Kawasaki Disease Research Committee [14]. Patients were Han Chinese individuals 2 months to 9 years and 6 months of age. There were 82 males and 40 females with an average age at onset of 29.4 ± 22.2 months. Before hospital admittance, all patients had not received IVIG or aspirin treatment. To further investigate the relationship between CAL formation and *TM* gene polymorphisms, the KD group was divided into KD with coronary artery lesions (KD-CAL) and KD without coronary artery lesions (KD-WO) subgroups. All patients with KD received a series of imaging examinations, including pulse Doppler, two-dimensional and color flow echocardiogram, at least 3 times within 8 weeks from the onset of the illness. Echocardiographic follow-up was performed every 3 to 6 months in the first year for KD patients with abnormal coronary arteries and then once annually until the affected coronary arteries normalized. We used two-dimensional echocardiography to visualize the diameter of the left and right coronary arteries on the parasternal short-axis view of the aorta. In the echocardiogram, CAL is defined as the internal lumen diameter, of either the left or right coronary artery, of >3 mm in children <5 years of age and >4 mm in children ≥5 years of age; CAL can be also defined as the internal diameter of a segment that is at least 1.5 times that of an adjacent vessel or the coronary lumen is clearly irregular [15]. To investigate the relationship between intravenous immunoglobulin (IVIG) resistance and *TM* gene polymorphisms, we distinguished 17 patients resistant to IVIG as the IVIG-resistant group and 93 patients sensitive to IVIG as the IVIG-sensitive group. IVIG resistance was defined as a return of fever (rectal temperature > 38.5°C) associated with one or more of the initial symptoms that led to the diagnosis of KD within 2~7 days or even 2 weeks after the initial IVIG treatment. A total of 126 healthy controls were recruited from January 2012 to December 2016 in the Third Xiangya Hospital, Changsha, China. Healthy controls were 5 to 14 years of age and did not have any previous history of KD, infectious diseases, cardiovascular diseases, or rheumatic diseases. There is no statistically significant gender distribution difference between the KD patients and healthy controls (*P* > 0.05).

We collected blood samples from KD patients and the controls in disposable blood collection tubes (containing anticoagulant EDTA-Na_2_). Blood samples were immediately transferred to cryopreservation tubes and stored at −80°C. The study protocol was approved by the Ethics Review Committee of Medical Research Institute and Institutional Review Boards of the Medical Research Institute at the Third Xiangya Hospital.

### 2.2. DNA Extraction

Genomic DNA was isolated from peripheral blood leukocytes using a Wizard genomic DNA purification kit according to the manufacturer's instructions (Promega, Madison, WI, USA). Extracted DNA was detected by a NanoDrop ND-2000C Spectrophotometer (Thermo, USA).

### 2.3. Genotyping

The detection of genotypes of the *TM* C1418T polymorphism was tested with the polymerase chain reaction-sequence-based typing (PCR-SBT) method. The *TM* gene was amplified by polymerase chain reaction (PCR) using primers designed by the authors (forward: 5′-GCTACATCCTGGACGACGGTTTC-3′ and reverse: 5′-GGCAGAGGAGCGCCAAAAGC-3′) and a Thermal Cycler 9700 (Applied Biosystems, Foster City, CA, USA). PCR reactions were carried out in a total volume of 20 *μ*L containing 1.6 *μ*L of DNA, 10 *μ*L of 2x Taq Master Mix (CWBio, China), 0.8 *μ*L of forward primer (Sangon Biotech, China), 0.8 *μ*L of reverse primer (Sangon Biotech, China), and ddH_2_O (added to a final volume of 20 *μ*L). The following thermal cycling conditions were used: denaturing at 94°C for 5 minutes, followed by 35 cycles of denaturing at 94°C for 30 seconds, annealing at 66°C for 30 seconds, and extension at 72°C for 30 seconds, and then an extension at 72°C for 10 minutes. The amplification products were sent to a sequencing company (BioSune, China) for genetic sequencing. The sequences were read with the help of Chromas software.

### 2.4. Statistical Analysis

All statistical analyses were performed using SPSS software (version 17.0; SPSS Inc., Chicago, IL, USA). The *χ*^2^ test with 1 degree of freedom was used to perform the Hardy-Weinberg equilibrium (HWE). The differences in genotype and allele frequencies between the two groups were evaluated using a *χ*^2^ test. The odds ratio (OR), along with its 95% confidence interval (95% CI), were estimated for associations between risk alleles and genotypes of KD. Two-sided *P*values < 0.05 were considered statistically significant.

## 3. Results

### 3.1. Association of TM Gene C1418T Polymorphism with Susceptibility to KD

**Figure 1:**
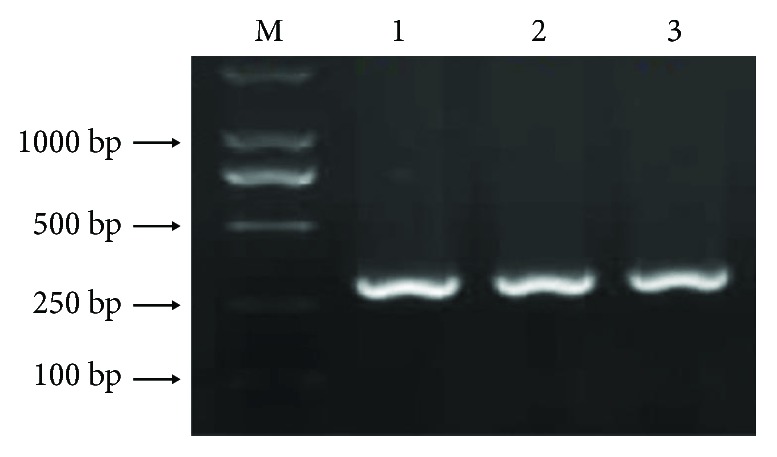
Agarose electrophoresis charts of *TM* gene PCR products. M: DNA marker. Lanes 1–3: *TM* gene PCR products (amplification length: 324 bp).

**Figure 2:**
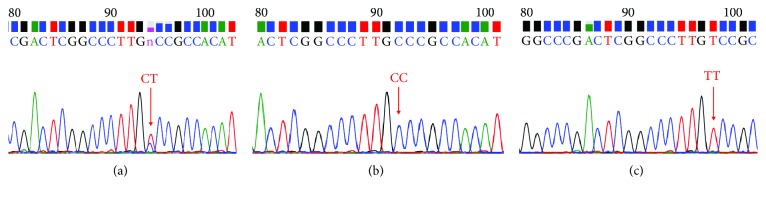
The genotype test at locus C1418T in the *TM* gene. (a) CT genotype; (b) CC genotype; (c) TT genotype.

The 324 bp amplification products were separated using gel electrophoresis (Figure 1). The PCR sequence-based typing results are shown in Figure 2. The genotypes of the single-nucleotide polymorphism were in line with the HWE for cases and controls (*χ*^2^ = 1.05, 2.46, *P* > 0.05). As shown in Tables 1–3, the distribution of genotypes (CT, TT) in the KD group was different from that in the control group (*χ*^2^ = 12.091, *P* = 0.002); carriers of allele T had a 1.852 times higher risk of KD than that of noncarriers (*χ*^2^ = 8.862, *P* = 0.001, OR = 2.356, 95% CI = 0.246–0.689).

### 3.2. Association of TM Gene C1418T Polymorphism with CAL Formation

As shown in Table 1, there were no significant differences in frequencies of genotypes (CC, CT, and TT) and alleles (C, T) between the KD-CAL group and the KD-WO group (*P* > 0.05).

### 3.3. Association of TM Gene C1418T Polymorphism with IVIG Resistance

As shown in Table 1, there were no significant differences in frequencies of genotypes (CC, CT, and TT) and alleles (C, T) between the IVIG-resistant group and the IVIG-sensitive group (*P* > 0.05).

## 4. Discussion

The *TM* gene is located on chromosome 20p11.2 and contains a single exon and no introns. TM is expressed primarily on the luminal surface of vascular endothelial cells and consists of 557 amino acids (aa): an N-terminal lectin-like module (aa 1–154), a hydrophobic region (aa 155–222), six epidermal growth factor- (EGF-) like modules (aa 223–462), a serine- and threonine-rich region (aa 463–497), a single transmembrane segment (aa 498–521), and a short cytoplasmic tail (aa 522–557). There are 6 EGF-like repeats at the EGF-like domain of the extracellular region, and the last 3 repeats are functionally important for protein C activation and thrombin binding [16]. The enzymatic cleavage from the cell surface produces soluble thrombomodulin (sTM) in the plasma. TM has at least three major anticoagulant characteristics: (1) it catalyzes thrombin activation of protein C; (2) it alters thrombin substrate specificity, which leads to inhibition of thrombin-mediated clotting, platelet activation, and procoagulant factors (V, VIII, XI, and XIII); and (3) it plays a significant role in the inhibition of thrombin by antithrombin. The increasing level of sTM can be detected in various kinds of endothelial cell injuries, such as plasma, urine, and joint synovial fluid [17]. The normal vascular endothelium can secrete anticoagulants (TM, heparin, tissue plasminogen activator, etc.) to maintain local blood coagulation, fibrinolysis balance, and blood flow. We know that TM plays an important role in anticoagulation. In addition, TM can exert its anti-inflammatory effect via the activated protein C- (APC-) independent pathway and APC-dependent pathway [18]. The *TM* C1418T polymorphism, which encodes for the replacement of Ala455 by Val455 in the *TM* gene, has been well described in previous studies. This polymorphism is located in the coding region of the *TM* gene, which is responsible for thrombin binding and protein C activation, signifying its potential role in regulating thrombomodulin function [16].

The *TM* C1418T polymorphism has been widely found to be associated with cardiac-cerebral vascular diseases. Wang and Dong [19] conducted a meta-analysis study to uncover the association between two SNPs in the *TM* gene, −33G/A and Ala455Val (C1418T), and coronary artery disease (CAD), and they reported a significant association of the two loci of the gene polymorphism with CAD. Lobato et al. [20] found that allele T of the locus C1418T in the *TM* gene is independently associated with increased long-term mortality risk following coronary artery bypass grafting (CABG) and significantly improved the classification ability of traditional postoperative mortality prediction models. A study from north India showed that the *TM* C1418T polymorphism is an independent predictor of acute myocardial infarction (AMI) [21].

According to previous studies, there is a close relationship between the development of KD and endothelial cell injury, a hypercoagulable state, and thrombosis tendency [22]. Thus, *TM* gene polymorphisms may be associated with KD, but there has been no published research in this field.

The present study results illustrate that there were significant differences in genotype frequencies of CC, CT, and TT between the KD group and the control group. In addition, carriers with genotype CT had a 2.356 times higher risk of KD than that of noncarriers, and carriers with allele T had a 1.852 times higher risk of KD than that of noncarriers. However, there were no significant differences in frequencies of genotypes (CC, CT, and TT) or alleles (C, T) between the KD-CAL and KD-WO groups and IVIG-resistant and IVIG-sensitive groups. These findings suggest that the genotype CT and allele T may be susceptible factors to KD because of the significant correlation between the *TM* C1418T polymorphism and KD. However, the *TM* C1418T polymorphism was not found to be associated with CAL formation or IVIG resistance. There are several possible reasons for this finding: the sample size was not sufficient to insure the accuracy of the results; C1418T may not be the responsible locus for the association between the *TM* gene polymorphism, CAL formation, and IVIG resistance; or there is indeed no specific relationship between the *TM* gene polymorphism, CAL formation, and IVIG resistance. Hence, further larger studies with more information could verify our findings.

In conclusion, our study illuminated that the *TM* C1418T polymorphism was significantly associated with the risk of KD.

## Figures and Tables

**Table 1:** Frequencies of *TM* genotypes in the study subjects.

Groups	*n*	Genotypes [*n* (%)]			*χ* ^2^	*P*
		CC	CT	TT		
Control	126	83 (65.9)	35 (27.8)	8 (6.3)	12.091	0.002
KD	122	54 (44.3)	58 (47.5)	10 (8.2)		
KD-WO	92	42 (45.7)	44 (47.8)	6 (6.5)	1.323	0.557
KD-CAL	30	12 (40.0)	14 (46.7)	4 (13.3)		
IVIG-sensitive	93	37 (39.8)	48 (51.6)	8 (8.6)	3.639	0.164
IVIG-resistant	17	11 (64.7)	5 (29.4)	1 (5.9)		

**Table 2:** Frequencies of *TM* alleles in the study subjects.

Groups	*n*	Alleles [*n* (%)]		*χ* ^2^	*P*
		C	T		
Control	126	201 (79.8)	51 (21.2)	8.862	0.003
KD	122	166 (68.0)	78 (32.0)		
KD-WO	92	128 (69.5)	56 (30.5)	0.547	0.460
KD-CAL	30	38 (63.3)	22 (36.7)		
IVIG-sensitive	93	122 (65.6)	64 (34.4)	2.459	0.780
IVIG-resistant	17	27 (79.4)	7 (20.6)		

**Table 3:** Genotype and allele frequencies of the *TM* Gene in controls and patients with KD.

Genotypes/alleles	Controls [*n* (%)]	KD [*n* (%)]	*χ* ^2^	*P*	OR	95% CI
CC	83 (65.9)	54 (44.3)	11.708	0.001	0.411	0.246–0.687
CT	35 (27.8)	58 (47.5)	9.504	0.001	2.356	1.390–3.993
TT	8 (6.3)	10 (8.2)	0.100	0.752	1.317	0.502–3.457
C	201 (79.8)	166 (68.0)	8.263	0.04	0.54	0.359–0.812
T	51 (20.2)	78 (32.0)	8.263	0.04	1.852	1.231–2.786

## Data Availability

The data used to support the findings of this study are available from the corresponding author upon request.

## References

[1] Yim D., Curtis N., Cheung M., Burgner D. (2013). Update on Kawasaki disease: epidemiology, aetiology and pathogenesis. *Journal of Pediatrics and Child Health*.

[2] Kim K. Y., Kim D. S. (2016). Recent advances in Kawasaki disease. *Yonsei Medical Journal*.

[3] Singh S., Vignesh P., Burgner D. (2015). The epidemiology of Kawasaki disease: a global update. *Archives of Disease in Childhood*.

[4] Singh S., Bhattad S., Gupta A., Suri D., Rawat A., Rohit M. (2016). Mortality in children with Kawasaki disease: 20 years of experience from a tertiary care centre in North India. *Clinical and Experimental Rheumatology*.

[5] Kato H., Sugimura T., Akagi T. (1996). Long-term consequences of Kawasaki disease. A 10- to 21-year follow-up study of 594 patients. *Circulation*.

[6] Makino N., Nakamura Y., Yashiro M. (2015). Descriptive epidemiology of Kawasaki disease in Japan, 2011–2012: from the results of the 22nd nationwide survey. *Journal of Epidemiology*.

[7] Uehara R., Yashiro M., Nakamura Y., Yanagawa H. (2011). Parents with a history of Kawasaki disease whose child also had the same disease. *Pediatrics International*.

[8] Arj-Ong S., Thakkinstian A., McEvoy M., Attia J. (2010). A systematic review and meta-analysis of tumor necrosis factor *α*-308 polymorphism and Kawasaki disease. *Pediatrics International*.

[9] Li Z., Han D., Jiang J., Chen J., Tian L., Yang Z. (2017). Association of PECAM-1 gene polymorphisms with Kawasaki disease in Chinese children. *Disease Markers*.

[10] Weng K. P., Hsieh K. S., Hwang Y. T. (2010). IL-10 polymorphisms are associated with coronary artery lesions in acute stage of Kawasaki disease. *Circulation Journal*.

[11] Mikacenic C., Hahn W. O., Price B. L. (2015). Biomarkers of endothelial activation are associated with poor outcome in critical illness. *PLoS One*.

[12] Azme A. A., Shome D. K., Salem A. H., Fadhli S. A., Bannay R. A., Jaradat A. (2015). Thrombomodulin gene proximal promoter polymorphisms in premature acute coronary syndrome patients in Bahrain. *Blood Coagulation & Fibrinolysis*.

[13] Cole J. W., Roberts S. C., Gallagher M. (2004). Thrombomodulin Ala455Val polymorphism and the risk of cerebral infarction in a biracial population: the stroke prevention in young women study. *BMC Neurology*.

[14] Ayusawa M., Sonobe T., Uemura S. (2005). Revision of diagnostic guidelines for Kawasaki disease (the 5th revised edition). *Pediatrics International*.

[15] (2005). Guidelines for diagnosis and management of cardiovascular sequelae in Kawasaki disease. *Pediatrics International*.

[16] Anastasiou G., Gialeraki A., Merkouri E., Politou M., Travlou A. (2012). Thrombomodulin as a regulator of the anticoagulant pathway: implication in the development of thrombosis. *Blood Coagulation & Fibrinolysis*.

[17] Strijbos M. H., Rao C., Schmitz P. I. (2008). Correlation between circulating endothelial cell counts and plasma thrombomodulin levels as markers for endothelial damage. *Thrombosis and Haemostasis*.

[18] Carnemolla R., Patel K. R., Zaitsev S., Cines D. B., Esmon C. T., Muzykantov V. R. (2012). Quantitative analysis of thrombomodulin-mediated conversion of protein C to APC: translation from *in vitro* to *in vivo*. *Journal of Immunological Methods*.

[19] Wang H., Dong P. (2015). Thrombomodulin −33G/A and Ala455Val polymorphisms are associated with the risk of coronary artery disease: a meta-analysis including 12584 patients. *Coronary Artery Disease*.

[20] Lobato R. L., White W. D., Mathew J. P. (2011). Thrombomodulin gene variants are associated with increased mortality after coronary artery bypass surgery in replicated analyses. *Circulation*.

[21] Dogra R., Das R., Ahluwalia J., Kumar R. M., Talwar K. K. (2013). Association of thrombomodulin gene polymorphisms and plasma thrombomodulin levels with acute myocardial infarction in north Indian patients. *Clinical and Applied Thrombosis/Hemostasis*.

[22] Yahata T., Suzuki C., Yoshioka A., Hamaoka A., Ikeda K. (2014). Platelet activation dynamics evaluated using platelet-derived microparticles in Kawasaki disease. *Circulation Journal*.

